# Phenotypic and genetic characterization of a family carrying two Xq21.1-21.3 interstitial deletions associated with syndromic hearing loss

**DOI:** 10.1186/s13039-015-0120-0

**Published:** 2015-03-20

**Authors:** Sandra Iossa, Valerio Costa, Virginia Corvino, Gennaro Auletta, Luigi Barruffo, Stefania Cappellani, Carlo Ceglia, Giovanni Cennamo, Adamo Pio D’Adamo, Alessandra D’Amico, Nilde Di Paolo, Raimondo Forte, Paolo Gasparini, Carla Laria, Barbara Lombardo, Rita Malesci, Andrea Vitale, Elio Marciano, Annamaria Franzè

**Affiliations:** DMMBM, Università di Napoli “Federico II”, Naples, Italy; Ceinge Biotecnologie Avanzate, Naples, Italy; IGB “A. Buzzati Traverso”, CNR, Naples, Italy; Istituto di Audiologia, Dipartimento di Neuroscienze, Scienze Riproduttive e Odontostomatologiche, Università di Napoli “Federico II”, Naples, Italy; Institute for Maternal and Child Health - IRCCS “Burlo Garofolo”, Trieste, Italy; Dipartimento di Oftalmologia, Università di Napoli “Federico II”, Naples, Italy; University of Trieste, Trieste, Italy; Dipartimento di Scienze Biomediche Avanzate, Università di Napoli “Federico II”, Naples, Italy; Dipartimento di Oftalmologia Pediatrica, Università di Salerno, Salerno, Italy; Dipartimento di Scienze Motorie e del Benessere, Università di Napoli “Parthenope”, Naples, Italy

**Keywords:** Choroideremia, Hypotonia, Interstitial deletions, Intellectual disability, X-linked hearing impairment

## Abstract

**Background:**

Sensorineural hearing impairment is a common pathological manifestation in patients affected by X-linked intellectual disability. A few cases of interstitial deletions at Xq21 with several different phenotypic characteristics have been described, but to date, a complete molecular characterization of the deletions harboring disease-causing genes is still missing. Thus, the aim of this study is to realize a detailed clinical and molecular analysis of a family affected by syndromic X-linked hearing loss with intellectual disability**.**

**Results:**

Clinical analyses revealed a very complex phenotype that included inner ear malformations, vestibular problems, choroideremia and hypotonia with a peculiar pattern of phenotypic variability. Genomic analysis revealed, for the first time, the presence of two close interstitial deletions in the Xq21.1-21.3, harboring 11 protein coding, 9 non-coding genes and 19 pseudogenes. Among these, 3 protein coding genes have already been associated with X-linked hearing loss, intellectual disability and choroideremia.

**Conclusions:**

In this study we highlighted the presence of peculiar genotypic and phenotypic details in a family affected by syndromic X-linked hearing loss with intellectual disability. We identified two, previously unreported, Xq21.1-21.3 interstitial deletions. The two rearrangements, containing several genes, segregate with the clinical features, suggesting their role in the pathogenicity. However, not all the observed phenotypic features can be clearly associated with the known genes thus, further study is necessary to determine regions involved.

**Electronic supplementary material:**

The online version of this article (doi:10.1186/s13039-015-0120-0) contains supplementary material, which is available to authorized users.

## Background

Sensorineural hearing impairment (SNHI) can be considered the most frequent form of children’s hearing loss (1/1000 born) and about 60% of the cases can be ascribed to genetic causes [1]. Most of SNHI forms are non-syndromic, representing 70% of all cases, even though at least 400 known syndromes have been described. Intellectual disability (ID) is a disorder that sometimes co-occurs with sensorineural hearing impairment. It represents one of the major handicaps affecting 1% to 3% of the general population, with onset before age 18. It is more common in males because of the high incidence of mutations in genes located on the X chromosome [2]. To date, more than 80 chromosome X-linked ID genes (XLID) have been identified [3]. In most of them mutations give rise to syndromic ID forms, often accompanied by behavioral, somatic, metabolic or neurological disorders [4,5]. Syndromic forms can also result from contiguous gene deletions. In particular, deletions and rearrangements in the Xq21 have been linked to other complex syndromes including ID, deafness, choroideremia (CHM), seizures, and multiple congenital anomalies [6-11]. Smaller deletions lead to a milder clinical phenotype [9,10,12-16]. ID has been reported - in few cases - in association with deafness and CHM [17,18]. In these cases, the patients carried interstitial deletions within the Xq21. Schwartz et al. [19] mapped the deletion identified by Rosenberg and collaborators [18] at the Xq21.2-Xq21.31. More recently, a new 16 Mb deletion in the same region has been described [20]. Nonetheless, a complete molecular characterization of such deletions at the Xq21, harboring disease-causing genes, is still missing. In this study, we describe for the first time - to the best of our knowledge - a systematic clinical, genomic and molecular analysis of a family with a complex phenotype including syndromic congenital sensorineural X-linked hearing loss. Our analysis revealed the presence of two close interstitial deletions in the Xq21.1-21.3, harboring several protein-coding genes. We also discuss the potential role of the deletions in the described phenotype.

This paper is dedicated to the memory of the Pediatrician Dr. Alfredo Pisacane (1951–2012).

### Case presentation

**Figure 1:**
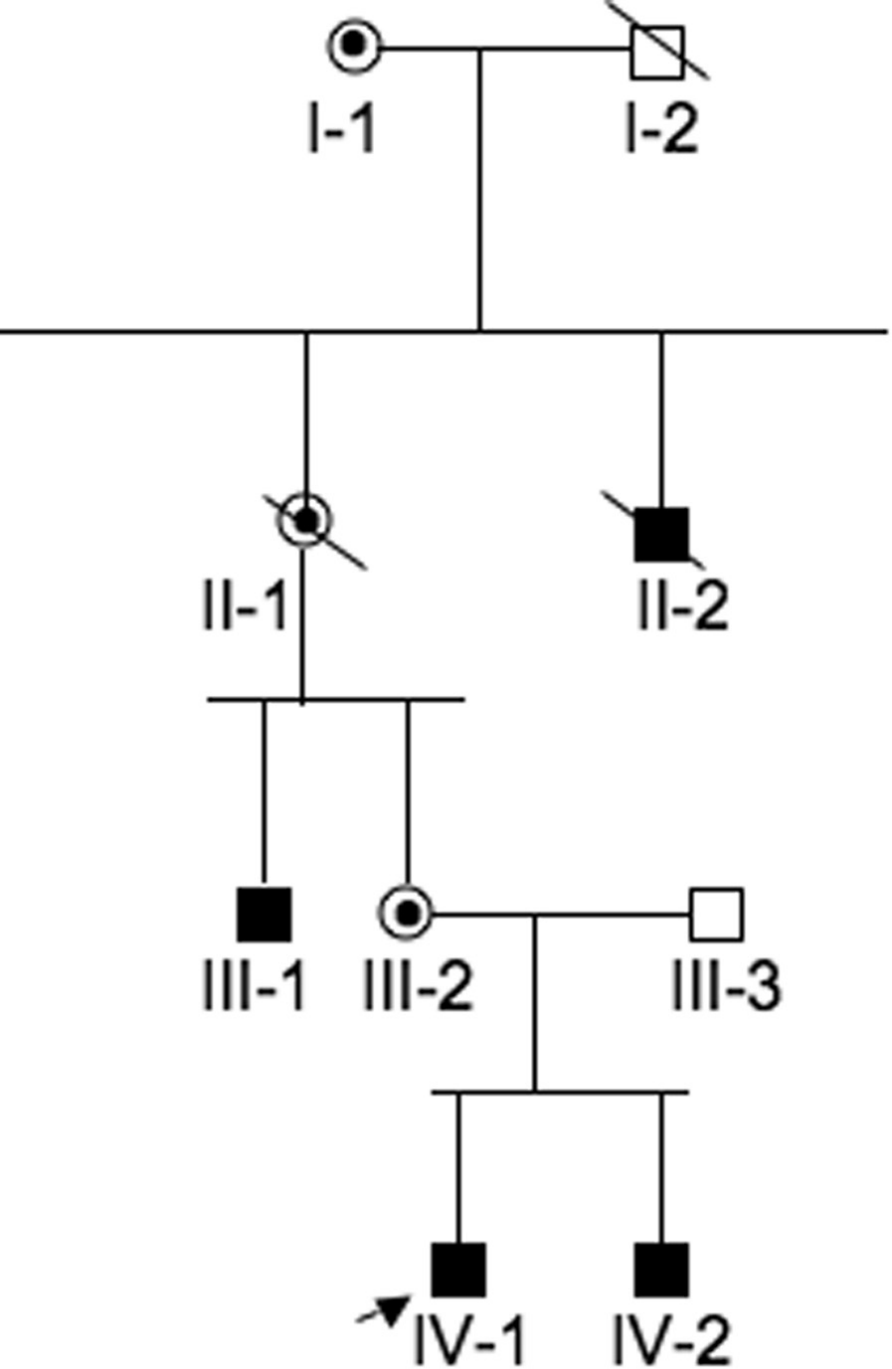
**Pedigree of the affected Italian family.** Squares and circles symbolize males and females, respectively. Unblackened and blackened symbols denote unaffected and affected individuals, respectively. Circles with a dot inside denote carrier females. Diagonal lines denote deceased individuals. The arrow denotes the proband.

A four-generation Italian family (Figure 1) with an X-linked syndromic form of hearing impairment was studied. The proband (IV-1) and his brother (IV-2) had congenital bilateral hearing SNHL that had been assessed by our group a few months after birth as described in Chinetti et al. [21]. At 2 years of age both individuals showed psycho-neuro-motor disabilities too, with different degrees of severity. In the course of this study, several other clinical signs, summarized in Table 1, were observed. In detail: proband (IV-1) born in 2006, was diagnosed with bilateral severe hearing SNHL at 5 months of age. At 2 years of age, he was diagnosed with psycho-neuro-motor disability. He presented also trunk and limb hypotonicity, poor motor coordination, and was unable to walk unsupported. He was fitted with hearing aids by 6 months of age, but since January 2012 has refused to use them. At present, the ID is profound with no evolutive process. Comprehension is very poor; he recognizes only the use and function of simple and familiar objects. Verbal language is absent and he use a mimic sign language with motor stereotypies. He presents a psychotic behavioral and relational disorder. He presents balance problems and flat feet too. Proband’s brother (IV-2) born in 2007, was diagnosed at 5 months with a SNHL similar to the proband. Subsequently, at about 2 years of age, he was also diagnosed with trunk and limbs hypotonicity and psycho-neuro-motor disability. At present, he presents a phenotype similar to that of his brother but in a much milder form. Like his brother, he recognizes only the use and function of simple and familiar objects. Verbal language and comprehension, in spite of constant hearing aid use, is poor, but better than that of his elder brother.

**Table 1:** **Clinical details for affected subjects**

	**IV-1**	**IV-2**	**III-1**	**II-2**
**Age (years)**	8	7	35	-
**- SNHL**	Congenital/Severe	Congenital/Severe	Congenital/Profound	Congenital/Profound
**- Tympanometry**	Normal	Normal	Normal	Normal
**- Vestibular problems**	N.D.	N.D.	Mixed sufferance: central and peripheral	N.D.
**- ID**	Very Severe	Severe	Moderate	Severe
**- Age of hearing aids fitting**	6 months	6 months	9 years	N.D.
**- Constant use of hearing aids**	No	Yes	Yes	N.D.
**- Comprehension**	Very poor: recognition only of the use and function of simple and familiar objects. Psychotic behavioral and relational disorder	Poor: recognition only of the use and function of simple and familiar objects	Good	N.D.
**- Verbal language**	Absent: use of a mimic sign language	Poor	Concrete and fluent	N.D.
**- Trunk and limbs hypotonicity**	Present	Present, but milder than IV:1	Absent	N.D.
**- Motor stereotypies**	Present	Present	Absent	N.D.
**- CHM evaluation**	N.D.	N.D.	Alteration typical of choroideremia	N.D.
**- Brain MRI/CT**	Normal	Normal	Mega cisterna ampia. Cerebral cysticercosis	N.D.
**- Ear MRI/CT**	Inner ear strongly altered. Small oval window	Inner ear strongly altered. Small oval window	Inner ear strongly altered. Dysplastic fine irregularity of the profile of the semicircular canals	N.D.
**- Large forehead, down turned rimae palpebrarum and large ears**	Present	Present	Not prominent	N.D.
**- Flat feet**	Present	Present	Present	N.D.

N.D.: not disposable.

Subject III-1: Born in 1969; he was diagnosed in infancy with bilateral profound SNHL and psycho-neuro-motor disability. The intellectual disability is of medium severity: he is sufficiently able to perform activities of daily living on his own. Neuropsychological tests determined a deficit that primarily occurs in short term memory for spatial material. He had been fitted with hearing aids for language development at nine years of age, and presents a simple concrete verbal language with a good verbal comprehension. Also this subject presents balance problems and flat feet but motor stereotypies are absent. Subject II-2: Born in 1947; he was diagnosed with profound bilateral hearing loss and very severe developmental delay and intellectual disability. He was not able to perform his activities of daily living unaided. Detailed clinical examinations had never been performed.

**Figure 2:**
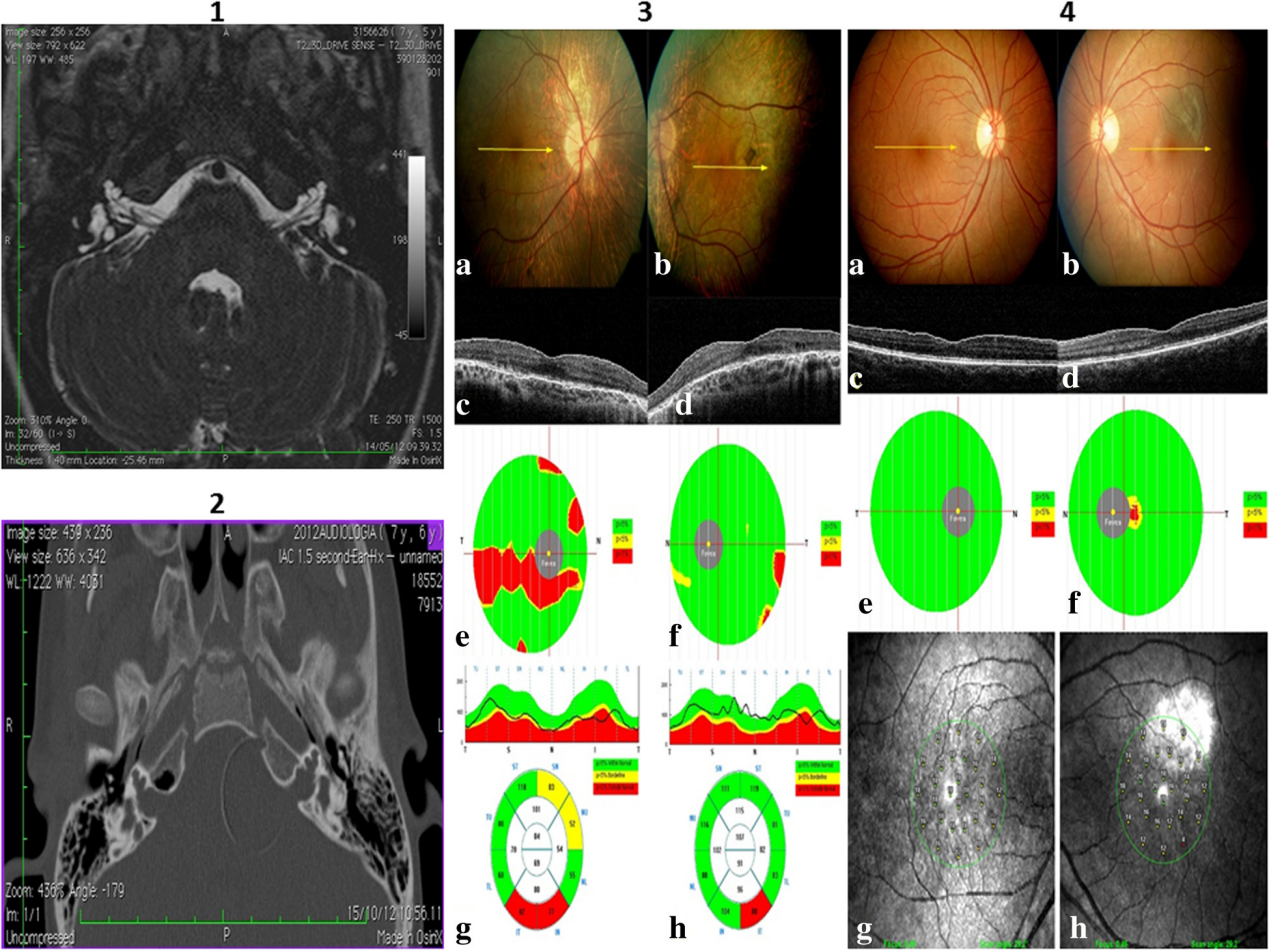
**Images for subject IV-1: 1) Axial MR TSE drive T2-weighted image.** Note the small bulbous dilatation of the lateral part of the IAC, with absence of the modiolus and of the bone at the fundus. The cochlea is dysmorphic and small in size. The vestibule is dilated bilaterally. Note also the dilatation of the vestibular acqueduct on the right side. **2)** Axial CT. The absence of the bone fundi of IAC and of the modiolus are better depicted on CT. Ophthalmic results: **3)** Male patient affected with choroideremia. Color fundus images (**a**, right eye; **b**, left eye) and cross sectional OCT B-scan (**c**, right eye; **d**, left eye) show diffuse atrophy of the retinal pigment epithelium and choriocapillaries and round retinal pigment changes at the posterior pole and midperiphery. Ganglion cell complex thickness shows a severe arciform thinning in **(e)** the right eye, while focal alterations are present in **(f)** the left eye. Retinal nerve fiber layer is reduced in the inferior quadrants in **(g)** the right and **(h)** left eye. **4)** Female patient carrier of the choroideremia gene. Color fundus images (**a**, right eye; **b**, left eye), cross sectional OCT B-scan (**c**, right eye; **d**, left eye), ganglion cell complex thickness map (**e**, right eye; **f**, left eye) and microperimetry (**g**, right eye; **h**, left eye) are normal. A choroidal nevus is visible at the posterior pole in the left eye.

The evaluation of patients by computed tomography (CT) and magnetic resonance imaging (MRI) showed significant alterations of the inner ear apparatus, with some marked differences among affected individuals. Both CT and MRI of the siblings (IV-1, IV-2) and of the III-1 individual revealed a bulbous dilatation of the internal auditory canals (IAC), associated with the absence of the fundi of the bony plates separating the basal turn of cochlea and IAC. The modiolus was completely absent and the cochlea was dysmorphic and small, especially the medium and the apical turn. A mild dilatation of the vestibule and of the posterior semicircular canals was present in both the siblings, and proband (IV-1) also had a dilatation of the right vestibular aqueduct. In the two children, CT also revealed a small oval window that was not clearly appreciable in the individual III-1. Cochlear nerves were normal. The MRI analysis in the III-1 individual also showed a dysplastic fine irregularity in the profile of the semicircular canals, not appreciable in the other patients. Brain MRI of the subject III-1 revealed the presence of a mega cisterna magna and cerebral cysticercosis, while it did not reveal any morphological malformation in the other two patients (IV-1, IV-2) (Table 1). Figure 2 (panel 1, 2) reports MRI and CT of subject IV-1. Due to the inability to communicate and poor cooperation, it was possible to perform vestibular analysis only on individual III-1. The presence of alterations found in this subject suggest both central and peripheral suffering. Indeed, for this subject, the presence of a down nystagmus was shown in all positions, which was found to be persistent, symmetric and partially inhibited by visual fixation. Vestibulo-ocular reflex (VOR) was hypoactive bilaterally and slightly reduced by visual fixation. Finally, affected subjects presented some peculiar facial characteristics too: large forehead, down turned rimae palpebrarum and large ears.

No unaffected individuals or carrier females had mental, auditory or other significant problems related to those described in the affected individuals.

## Results

**Figure 3:**
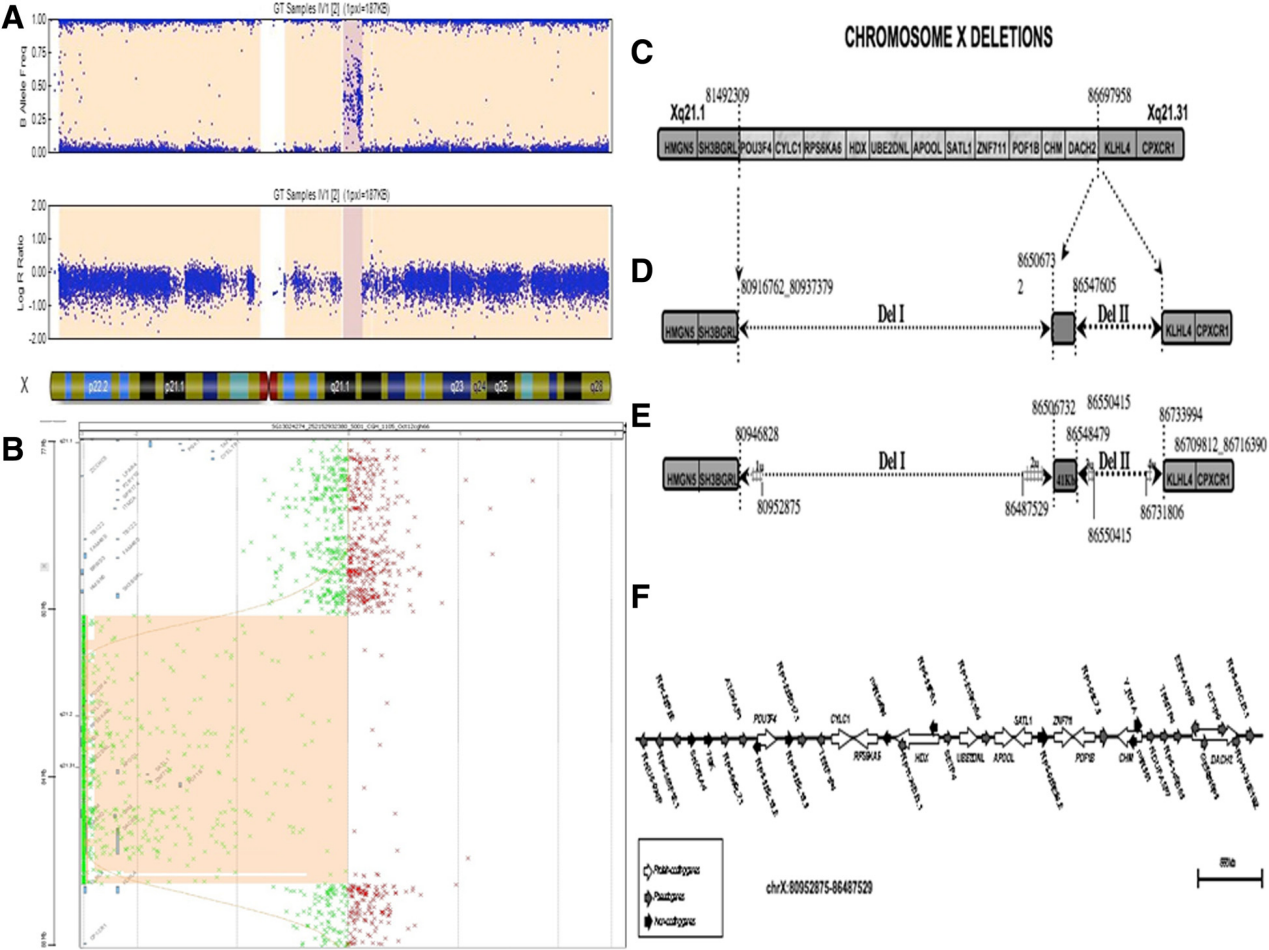
**Info on deleted region. A)** Graph of the marker-specific fluorescence (logR) and allele-specific fluorescence of SNPs on chromosome X in 1 of the patients. The deleted region is marked in dark pink. **B)** A-CGH profile of chromosome from the Agilent 1x1M array in the subject IV-2 shown that the deletion of 5,77 Mbp includes a small undeleted region of about 40 Kb. **C)** SNPs array results: deleted genes are reported in the light grey zone and undeleted genes in the dark grey zone. **D)** CGH array result: zones deleted are represented as dotted arrows. **E)** Refinement of breakpoint deletions results by PCR analysis. Gridded zone represents regions that remained undetermined. **F)** Schematic representation of different classes of genes identified in silico in the deleted region.The scale bar in the panel F refers to the entire genomic region that is deleted, whereas single genes or transcripts are not in scale. However, the genomic localization of each gene in this region is respected.

Clinical characteristics of the affected individuals are reported in Table 1. No abnormalities were indicated by previous high-resolution karyotype and fragile X molecular analyses. Thus, we first decided to perform a mutational screening in Gap junction beta-2 (*GJB2*), Gap junction beta-6 (*GJB6*) and Charcot-Marie-Tooth genes*:* phosphoribosyl pyrophosphate synthetase 1 (*PRPS1*) and Gap junction beta-1 *(GJB1*). These analyses did not reveal the presence of any mutation. Then, we carried out a linkage analysis on this family. Unfortunately, significant results were not obtained, possibly due to the presence of a large deleted region and the consequent absence of specific markers in that region. Finally, we used a genome-wide approach, i.e. single nucleotide polymorphisms (SNPs) array analysis. Using this approach we were able to observe the presence of a deletion on X chromosome of about 5.2 Mb, spanning from the Xq21.1 to Xq21.31, from the nucleotide 81492309 to 86697958 (Figure 3A). Regarding protein-coding genes only, *in silico* analysis revealed that the deletion extends from POU class 3 homeobox 4 (*POU3F4*) to dachshund family transcription factor 2 (*DACH2*) genes (Figure 3C). Information about the coding genes mapping in the deleted region is reported in Additional file 1: Table S1_1 (see Supplementary data online S1.doc, section 1). Region-specific polymerase chain reaction (PCR) analyses on genomic DNA isolated from affected (III-1, IV-1, IV-2) and healthy carrier (III-2) individuals confirmed the absence of all these genes, and the presence of SH3 domain binding glutamate-rich protein like (*SH3BGRL*) and kelch-like family member 4 (*KLHL4*) genes mapping proximally to the deletion. Then, to better refine the boundaries of the deleted region we used comparative genomic hybridization (CGH) arrays. Using this approach we confirmed the presence of a deletion of about 5.77 Mb, (*chrX.hg19:g.(80,916,762_80,955,540)_(86,709,812_86,736,428)del*). Interestingly, we also discovered that inside the deleted region in the proband (*chrX.hg19:g.(80,916,762_80,937,379)_(86,709,812_86,716,390)del*.), a specific genomic region from the nucleotide 86,506,732 to 86,547,605 was not deleted (Figure 3B). This analysis indicated the presence of two close interstitial deletions, named “Del I” (of about 5.57 Mb) and “Del II” (of about 162 Kb). These two deletions are spaced out by a region of about 41 Kb (Figure 3D). The presence of this region was confirmed by PCR assays (supplementary data online S1.doc, section 2: primers DelF2/DelR2 in Additional file 1: Table S1_2) carried out in all of the affected individuals (data not shown). Starting from these results, a PCR-based assay was used to further refine the two deletions’ breakpoints. Given the high content of repeated regions encompassing the deleted portion of X chromosome, the exact sequence of some genomic regions (indicated in Figure 3E as 1u-4u) is still undetermined. However, these analyses allowed us to determine that Del I consists of 5.56 Mb (with an ambiguity of about 25 Kb as shown in Figure 3E, 1u + 2u) and Del II consists of 185.5 Kb (with an ambiguity of about 4 Kb, 3u + 4u). Browsing the main public genomic databases, we observed that the deleted region Del I contains several coding and non-coding genes (Figure 3F). In particular, the *in silico* analysis revealed that the patients carrying Del I completely lack the genomic region encompassing 11 protein-coding, 9 non-coding genes and 19 pseudogenes. Within this region, 3 protein-coding genes (*CHM, Pou3F4* and *ZNF711*) have already been associated with syndromic congenital sensorineural X-linked hearing impairment, ID and CHM. The other deleted genes are not associated with the observed phenotypes, indicating them as potential contributors to the complex phenotype of affected individuals. No genes are annotated in the 41 Kb region or in Del II. By reverse transcriptase PCR (RT-PCR) on RNA isolated from peripheral blood mononuclear cells (PBMCs), we experimentally confirmed that in the III-1 individual Del I and Del II do not alter the expression of the two genes located in close proximity of the 5′ and 3′ boundaries of the identified deletions: *SH3BGRL* and *KLHL4* (Supplementary data online Additional file 2: Figure S1).

Prior to this study, no significant visual disturbance had been noticed in any of the family subjects. Therefore, as also the *CHM* gene - involved in choroideremia onset - was deleted, we carried out a detailed ophthalmological study on individuals III-1 and III-2. Clinical alterations typical of a CHM affected individual were observed for patient III-1 (Figure 2, panel 3), whereas a mild phenotype, typical of carrier individuals was reported in patient III-2 (Figure 2, panel 4).

## Discussion

Sensorineural congenital hearing loss, vestibular problems, intellectual disability, choroideremia, hypotonia and some peculiar facial dysmorphisms have been observed in the family members (Table 1). Few studies have reported some of the phenotypic characteristics (intellectual disability, hearing loss and choroideremia) here described [17,20]. However, a comprehensive clinical, genomic and molecular characterization has not yet been performed. It is particularly relevant to observe the phenotypic variability in the family subjects reported in this study. In particular, the large difference in the degree of intellectual disability, which is more pronounced for proband (IV-1) compared to his brother (IV-2) and above all to their uncle (III-1). Indeed, even though individual III-1 did not undergo any treatment during his infancy he had a very milder phenotype compared to his relatives. Some differences among affected subjects have also been observed in the conformation of the inner ear and brain (Table 1).

From a molecular point of view this is the first report of two close deletions in the Xq21.1-21.3 regions: Del I and Del II (Figure 3D). This genomic region encompasses several genes, reported in detail in Figure 3C and Additional file 1: Table S1_1 (see Supplementary data online S1.doc, section 1). Three of them - *POU3F4*, *ZNF711* and *CHM* - are particularly significant for the clinical symptoms described in the affected individuals. *POU3F4* has a proven role in hearing loss and inner ear malformations [22], *ZNF711* has been associated with simple ID [23] and *CHM* with the choroideremia [24]. In the analyzed family, choroideremia symptoms are not particularly significant. Indeed, CHM has not been previously diagnosed in family members. CHM alterations have been determined only by instrumental ophthalmic exams (Table 1; Figure 2, panel 3 and 4). Our study also documented other peculiar phenotypic features, such as hypotonia and facial dysmorphism, in the family members. Interestingly, none of the genes present in the deletion described is clearly associated with these symptoms, as shown in Additional file 1: Table S1_1 (see Supplementary data online S1.doc, section 1).

However, although the other genes have not been associated with any of the observed pathological traits, we cannot exclude that the encoded proteins may interact with known disease-causing genes. Interestingly, browsing BioGRID - an online interaction repository - we observed that the protein encoded by *RPS6KA6* gene has more than 35 interactors, with a significant involvement in “central nervous system development” and “negative regulation of mesoderm development”. This observation suggests that some deleted genes may potentially encode proteins that interact, both directly and indirectly, with other proteins whose function is strictly correlated with the observed phenotypes. Other protein coding genes, not mapping to the deleted X chromosome region, may account for the unexpected pathological features. It indicates that further genome-wide analyses are needed to identify them. Moreover, our results highlight the presence of pseudogenes and of other non-coding genes in the deleted region. Notably, both pseudogenes and ncRNAs have been implicated in a novel regulatory networking (acting as ceRNA) and have been recently been indicated as relevant in neural cell plasticity and in neurodegenerative processes [25-28]. Therefore, we cannot exclude that the deletion of some processed pseudogenes, long ncRNAs (lncRNAs) and microRNAs (miRNAs) (Figure 3, panel F), may affect the expression of other genes, located elsewhere in the genome. Large-scale gene expression studies in these patients (by RNA-Sequencing) will clarify this crucial point. Del II deletion does not contain regulatory or coding sequences. This region may harbor a non-pathogenic copy number variation (CNV), which may have existed before the larger deletion arose. However, browsing of public CNV databases [29] excluded the presence of rearrangements encompassing the small deletions. However, the presence of still uncharacterized regions - with relevant coding or regulatory functions - in Del II cannot be excluded.

## Conclusions

In this study we identified two, previously unreported, Xq21.1-21.3 interstitial deletions in a family with a syndromic form of hearing loss with variable phenotype. The two rearrangements, segregate with the clinical features in affected subjects, suggesting their role in the pathogenicity. Not all the observed phenotypic features can be clearly associated to known genes. The presence of several pseudogenes and non-coding genes within the deleted region may correlate to the variable phenotype observed, and potentially affect unexplored molecular pathways or involve yet underestimated epigenetic mechanisms. So, further studies are necessary to obtain a more detailed genotype-phenotype correlation.

Data herein described confirm that this region of the X chromosome is characterized by increased proneness to chromosome breakage.

## Methods

Affected individuals IV-1, IV-2 and III-1 were analyzed by CT scan of ears and brain without contrast medium and by MRI of brain and ears (Philips 1.5 T). Gadolinium injection was subsequently required because of a suspected associated brain cysticercosis in individual III-1. Vestibular examination was performed with videonystagmoscopy (VNS) and included testing for spontaneous and positional nystagmus (head-hanging, left and right lateral supine positions) with and without visual fixation; testing for VOR generated by passive head rotation in the horizontal plane; VOR suppression test. Ophthalmic examination included best-corrected visual acuity (BCVA) by Snellen chart, intraocular pressure tonometry, anterior segment examination and ophthalmoscopy. Combined spectral domain optical coherence tomography/microperimetry system (SD-OCT, OCT/SLO, OPKO-OTI, Miami, FL) was used to obtain fundus-related microperimetry [30,31]. For fundus-related microperimetry, we used the Polar 3 testing pattern, a stimulus size Goldmann III test spot (Ø = 26 arc/min), stimulus intensity between 16 and 0 dB (10–400 asb). The RTVue-100 SD-OCT device (Optovue, Inc., Fremont, CA, USA) was used to obtain cross sectional B-scans of the posterior pole, retinal thickness in the 3 mm diameter central ring of the ETDRS map (central macular thickness, CMT), thickness of the retinal ganglion cell complex (GCC) and retinal nerve fiber layer (RNFL). RTVue-100 incorporates a GCC scan mode to measure inner macular retinal layer thickness, consisting of retinal nerve fiber layer, ganglion cell layer and inner plexiform layer, centered on the fovea and covering the central macula. For the peripapillary RNFL measurements, the RNFL exam protocol was used for scan acquisition, which was measured automatically at a diameter of 3.45 mm around the center of the optic nerve. For mutational screening genomic DNA was extracted by conventional salt precipitation protocols from peripheral blood samples collected in EDTA-containing tubes or from buccal swabs (Gentra System, DNA Isolation Kit). Amplified PCR fragments of the exon 1 and 2 of the *GJB2* gene were directly sequenced as reported elsewhere [32]. The two hearing loss-related deletions of the *GJB6* gene, del (*GJB6*-D13S1830) and del (*GJB6*-D13S1854), were screened using a combined detection procedure [33]. For Charcout-Marie-Tooth genes (*PRPS1*, *GJB1*) see Supplementary data online S1.doc, section 3). SNPs analysis was performed on the genomic DNA isolated from the patient and his relatives. All samples were genotyped using the Illumina HumanCytoSNP-12 BeadChip using the standard protocol. All samples were then checked for mendelian errors using Pedcheck and Pedstat [34]. Linkage analysis was performed using an X-linked parametric model with minx and complete penetrance [35]. High resolution a-CGH analysis was performed on the genomic DNA of the patient and his relatives. DNA digestion, labeling and hybridization were performed according to the manufacturer’s protocols. DNA specimens were analyzed with the Human Genome CGH Microarray kit 4x180K (Agilent Technologies, Santa Clara, CA), with an average space of 13 Kb and allowing an average resolution of 25 Kb. The proband’s genomic DNA was further analyzed with the Human Genome CGH Microarray kit 1xM (Agilent Technologies, Santa Clara, CA), with an average space of 2,1 Kb. Microarrays were scanned on the Agilent G2565CA scanner and image files were quantified using Agilent’s Feature Extraction software (V10.10.1.1); data were visualized with Agilent’s Genomic Work Bench Standard Edition (V6.5.0.58). Primer pairs for PCR analyses were designed using the Primer3 software [36]. The primer pairs obtained were checked for non-specific hybridizations in other genome regions using the National Center for Biotechnology Information (NCBI) Basic Local Alignment Search Tool (BLAST) [37]. The different amplified fragments have a variable length due to the need to not amplify regions of the X chromosome frequently repeated in the entire genome, in particular, given the high presence of repeated sequences dispersed in the genomic region analyzed, we took advantage of the RepeatMask track of the USCS Genome Browser [38] to design oligonucleotide pairs to validate the deletions. Primer sequences and PCR conditions used in this study are reported in supplementary data online S1.doc, section 2). Total RNA was isolated from PBMCs. PBMCs were separated by Ficoll gradient centrifugation, placed in Trizol (Invitrogen), and stored at −80°C according to the manufacturer’s. RNA integrity and concentration were evaluated by using the Nanodrop (Thermo Scientific). The quality of RNA from each sample was considered good if the 260/280 ratio was in between 1.8 and 2.0.

RT-PCR reaction was carried out by reverse transcription of total RNA using High Capacity cDNA Reverse Transcription Kit (Life Technologies). Primer sequences and PCR conditions on cDNA are reported in supplementary data online S1.doc, section 4). An extensive search for the presence of transcripts mapping within the genomic region encompassing the deletions has been performed retrieving the main genomic databases. Gene annotation tracks of RefSeq (release 62), Ensembl (version 74) and GENCODE (v19) have been loaded and visualized in the USCS Genome Browser [38]. All the transcripts, generated by alternative splicing, of protein coding and non-coding genes have been considered for the analysis. Only for genes proximal to the breakpoint, peaks of ChIP-Seq data for transcription factors (TFs) binding (GENCODE) were analyzed. The presence of binding sites for specific transcription factors was evaluated by visual inspection of ChIP-Seq tracks on Genome Browser.

The expression patterns, for both the protein and - where available - the transcript, were evaluated by using The Human Protein Atlas database [39].

This research was approved by the Ethics Committee of University of Naples “Federico II” and written informed consent for participation in the study was obtained from all participants and from the parents of patients under 18 years of age before blood/saliva sampling.

## Additional files

Additional file 1:
**Methods.** PCR conditions and primers utilized for the different experiments realized in this study. Information on genes deleted. Additional file 2: Figure S1. Semiquantitative RT-PCR assay.
