# Supplementary material for: Phenotypic and genetic characterization of a family carrying two Xq21.1-21.3 interstitial deletions associated with syndromic hearing loss
Source: Mol Cytogenet. 2015 Mar 20;8:18. doi: 10.1186/s13039-015-0120-0 (PMC4376344; doi:10.1186/s13039-015-0120-0)
Supplement: Additional file 1: — Methods. PCR conditions and primers utilized for the different experiments realized in this study. Information on genes deleted. [file 13039_2015_120_MOESM1_ESM.doc]

**1. Info on deleted coding genes**

| **Gene** | **Info summary** | **Reference** |
| --- | --- | --- |
|  |  |  |
| POU3F4 (OMIM: [300039](http://omim.org/entry/300039)) | Encodes a neural transcription factors and plays a role in inner ear development. It is responsible for X-linked mixed hearing loss with stapes fixation and perilymphatic gusher (DFN3) (OMIM: [304400](http://www.ncbi.nlm.nih.gov/omim/304400)). | [22] |
| CYCL1 | Encodes a sperm head cytoskeletal protein. The encoded protein is associated with the calyx of spermatozoa and spermatids | [40] |
| RPS6KA6(OMIM: 300303) | Encodes the p90 ribosomal S6 kinase-4 (RSK4) that is still largely uncharacterized | [41] |
| HDX | Highly Divergent Homeobox involved in  [premature ovarian failure.](http://www.ncbi.nlm.nih.gov/pubmed/23441923) | [42] |
| UBE2DNL | Ubiquitin-Conjugating Enzyme E2D N-Terminal Like (Pseudogene) | [43] |
| Apool | Apolipoprotein O-like: a cardiolipin-binding constituent of the Mitofilin/MINOS protein complex determining cristae morphology in mammalian mitochondria. | [44] |
| SATL1 | Spermidine/spermine N1-acetyl transferase-like 1 | [40] |
| ZNF711 | Encodes a zinc-finger protein of unknown function. Associated with XLMR in two families with truncating ZNF711 mutations that had moderate intellectual disability  without consistent additional distinctive features. | [23] |
| POF1B (OMIM: 300603) | Associate with premature ovarian failure (OMIM: [300604](http://www.omim.org/entry/300604)) | [45] |
| CHM (OMIM: 300390) | Encodes REP1(RAB ESCORT PROTEIN 1)  a subunit of a 2-subunit RAB geranylgeranyl transferase, causative for chorideremia (omim: [303100](http://www.omim.org/entry/303100) | [24] |
| DACH2 (OMIM:300608) | Encode a protein similar to the Drosophila protein dachshund. Recently: Discovery of dachshund 2 protein as a novel biomarker of poor prognosis in epithelial ovarian cancer. | [46] |

**Table S1_1.** Info on known genes absent in affected subjects

- 1. **Additional references**

40. Gene [http://www.ncbi.nlm.nih.gov/gene]

41. Sun Y, Cao S, Yang M, Wu S, Wang Z, Lin X, et al. Basic anatomy and tumor biology of the RPS6KA6 gene that encodes the p90 ribosomal S6 kinase-4. Oncogene. 2013;32:1794–810.

42. Okten G, Gunes S, Onat OE, Tukun A, Ozcelik T, Kocak I. Disruption of HDX gene in premature ovarian failure. Syst Biol Reprod Med. 2013;59:218–22.

43. GeneCards [http://www.genecards.org].

44. Weber TA, Koob S, Heide H, Wittig I, Head B, van der Bliek A, et al. APOOL is a cardiolipin-binding constituent of the Mitofilin/MINOS protein complex determining cristae morphology in mammalian mitochondria. PLoS One. 2013;8:e63683.

45. Lacombe A, Lee H, Zahed L. Disruption of POF1B binding to nonmuscle actin filaments is associated with premature ovarian failure. Am J Hum Genet. 2006;79:113–9.

46. Nodin B, Fridberg M, Uhlén M, Jirström K: Discovery of dachshund 2 protein as a novel biomarker of poor prognosis in epithelial ovarian cancer. J Ovarian Res. 2012, 5:6

***2.* Deletion breakpoint refinement**

The PCR reactions were performed with Expand Long Template PCR System (Roche), with 300 ng of genomic DNA. Reactions were carried out on thermal cycler for 1 cycle of 94°C for 2 min, 10 cycles of 94°C for 10 s, touch down from 59°C to 54°C for 30 s and 68°C for 15 min, 25 cycles of 94°C for 15 s, 54°C for 30 s and 68°C for 15 min and final extension of 68°C for 7 min. The PCR products were displayed after electrophoresis on agarose gel.

| **NAME** | **PRIMER FORWARD (5’-3’)** | **PRIMER REVERSE (5’-3’)** |
| --- | --- | --- |
| **415786F/415786R** | AGCTTTCCTAATTGATCTTCTGT | TCGATATTTGTAAGTCAGGGGT |
| **D6F/D6R** | ATATACATTATTCTCAGCACCA | AGGATGATGCTGACCCATAA |
| **D9F/D9R** | ATATGAACAGACACTTCTCAAA | AGTTGTATATCCTTGAGGAATT |
| **D4F/D4R** | TCAACTTGTAAGTATCTTGAGT | ATGAGAATTGACTTCAAACTAG |
| **DelC1/DelC1** | CAGTCTAGTTCTTCCACGTT | TGACATCTTTAGTGCTTTCCT |
| **DelF2/DelR2** | CCATCTCTCCTTCTTATTACA | TGCTGGTAGATTTGTTTGATT |
| **DelF3/Del2-6R** | TTCCCTTTCTTGTTTCTTAGT | ATAGATCTTAAATGTTTTCAACA |
| **DelF3/Del2-7R** | TTCCCTTTCTTGTTTCTTAGT | GATGGGGCTGTTTTTTTCTT |
| **DelF3/Del2-1R** | TTCCCTTTCTTGTTTCTTAGT | ACAATAGCAGTTCAAATAGCAT |
| **Del2-10F/DelR3** | TACAATGCCTTTCAAGTTTACT | TGCTTATTATTCCCTCTTTTC |
| **Del2-9F/DelR3** | AAACTCTACAGTTTTACTTCTT | TGCTTATTATTCCCTCTTTTC |
| **Del2-3F/DelR3** | TTTGTTTTTATATTGTGTGCTCA | TGCTTATTATTCCCTCTTTTC |

Table S1_2. Primers for deletions breakpoint refinement

***3. GJB1* and *PRPS1* mutational analysis**

The PCR reactions were performed with AmpliTaq Gold (Applied Biosystem Roche), with 300 ng of genomic DNA. Reactions were carried out on thermal cycler for 1 cycle of 95°C for 10 min, 38 cycles of 95°C for 45 s, primers temperature melting for 45 s, 72°C for 60 s, and final extension of 72°C for 7 min. The PCR products were displayed after electrophoresis on agarose gel.

| **GENE NAME** | **PRIMER NAME** | **FORWARD 5’-3’** | **REVERSE 5’-3’** |
| --- | --- | --- | --- |
| ***GJB1*** | **GJB1** | CTTTCCTGCTACTGGCTCTT | TCTGCCTGCTGGGGATTACT |
| ***GJB1* PROMOTER** | **PROM1** | CTTGTCCCCACCCTCTAATAA | AGGTGGATGTGAAGAGGGGA |
|  | **PROM2** | GTCCTCTTTCCTCTCCATATT | AAAACACCAGCCATGAAGCAA |
| ***PRPS1*** | **EX1** | AGAGCTACACCGAGGACCAA | TTCGCCTCACACTCCATCTT |
|  | **EX2** | TGTGGAACCTATGGATATGGA | AGGAAGTTGGTGCTTAGTCTTA |
|  | **EX3** | TACCATAGTGCCTTTAACATAGT | TCCCTATCTAACCACCTGAAA |
|  | **EX4** | TGATCTTGGCTGGGCTCTCT | ACTATATTTTCAACCCATGTGCTA |
|  | **EX5** | TCTTTAGTCCATTTCTTTTGTCTTA | TTACTTATCCCCTCAATTTGGT |
|  | **EX6** | TGCACCTTGATCTTGGACTTT | TCTTAGGCTCCATCTTCCAG |
|  | **EX7** | TGACAGGGAAACAGCACAGT | AGTTAAAGCTGCAAGGCCCA |

Table S1_3. Primers for mutational analysis in *GJB1* and *PRPS1* genes

**4- RTPCR and cDNA analysis**

RNA was used for reverse transcription reaction in a 20 μl of reaction mixture. The reaction conditions were as follows: 25°C for 10 min, 37°C for 120 min, followed by 85°C for 5 min.

The resulting cDNA was amplified using pairs of gene-specific oligonucleotides (Table S1_3). A heart cDNA was used to control the PCR reaction. PCR was performed with gene-specific primers using a My Taq DNA Polymerase (Bioline) kit. The reaction cycling conditions were as follows: 95°C for 2 min, followed by 35 cycles of 95°C for 20 s, 59°C for 20 s, 72°C for 20 s and final extension of 72°C for 7 min. PCR products were analyzed on agarose electrophoresis gel.

| **Gene** | **Forward 5’-3’** | **Reverse 5’-3’** |
| --- | --- | --- |
| **KLHL4** | AACTTCTGTGCAGTGATGACA | ATGGATCTTCTCTCAGGCAA |
| **SH3BGRL** | ATATTGCATCTTCCTCTGGCT | AACCTGTGGCTGGTCGACTA |

Table S1_4. Primers for cDNA analysis
