# Supplementary figures and images for: Phenotypic and genetic characterization of a family carrying two Xq21.1-21.3 interstitial deletions associated with syndromic hearing loss
Source: Mol Cytogenet. 2015 Mar 20;8:18. doi: 10.1186/s13039-015-0120-0 (PMC4376344; doi:10.1186/s13039-015-0120-0)

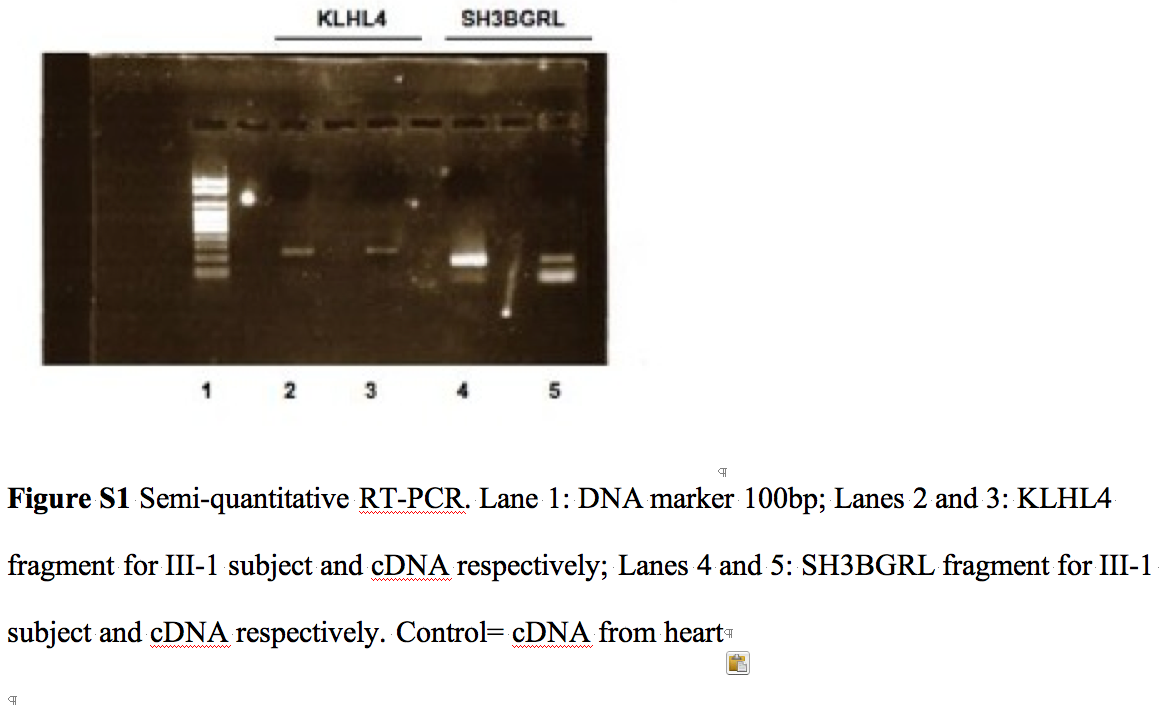

Supplement: Additional file 2: Figure S1. — Semiquantitative RT-PCR assay. [file 13039_2015_120_MOESM2_ESM.tiff]
